# Strategies to Assess Occupational Exposure to Airborne Nanoparticles: Systematic Review and Recommendations

**DOI:** 10.1016/j.shaw.2023.02.002

**Published:** 2023-02-28

**Authors:** Louis Galey, Sabyne Audignon, Patrick Brochard, Maximilien Debia, Aude Lacourt, Pierre Lambert, Olivier Le Bihan, Laurent Martinon, Sébastien Bau, Olivier Witschger, Alain Garrigou

**Affiliations:** 1 University Paris Nanterre, Department of Psychology, LAPPS, Team TE2O, Nanterre, France; 2 Univ. Bordeaux, INSERM, BPH, UMR1219, EPICENE Team, Bordeaux, France; 3 University Hospital of Bordeaux, Department of Environmental and Occupational Medicine, Bordeaux, France; 4 Department of Environmental and Occupational Health, School of Public Health, Centre de Recherche en Santé Publique (CReSP), Montreal, Québec, Canada; 5 CARSAT Aquitaine, Bordeaux, France; 6 INERIS, Verneuil en Halatte, France; 7 Service Parisien de Santé Environnementale, Laboratoire Amiante, Fibres et Particules, Ville de Paris, Paris, France; 8 INRS, Laboratoire de Métrologie des Aérosols, Vandoeuvre Lès Nancy, France

**Keywords:** Contextual information, Measurement strategy, Nanoparticles, Tiered approach, Work activity

## Abstract

In many industrial sectors, workers are exposed to manufactured or unintentionally emitted airborne nanoparticles (NPs). To develop prevention and enhance knowledge surrounding exposure, it has become crucial to achieve a consensus on how to assess exposure to airborne NPs by inhalation in the workplace. Here, we review the literature presenting recommendations on assessing occupational exposure to NPs. The 23 distinct strategies retained were analyzed in terms of the following points: target NPs, objectives, steps, “measurement strategy” (instruments, physicochemical analysis, and data processing), “contextual information” presented, and “work activity” analysis. The robustness (consistency of information) and practical aspects (detailed methodology) of each strategy were estimated. The objectives and methodological steps varied, as did the measurement techniques. Strategies were essentially based on NPs measurement, but improvements could be made to better account for “contextual information” and “work activity”. Based on this review, recommendations for an operational strategy were formulated, integrating the work activity with the measurement to provide a more complete assessment of situations leading to airborne NP exposure. These recommendations can be used with the objective of producing homogeneous exposure data for epidemiological purposes and to help improve prevention strategies.

## 1 Introduction

In workplace atmospheres, nanoparticles (NPs) are omnipresent. NPs used in manufacturing may be of natural or anthropic origin; they may also be produced unintentionally. When they are emitted during the production or handling of manufactured nanomaterials, NPs are generally designated by the term “engineered nanoparticles” (ENPs); more recently the concept of “nano-objects, agglomerates and aggregates (NOAA)” has emerged [1]. When emitted as an involuntary by-product of a process, they are generally referred to as “ultrafine particles” (UFPs). The processes leading to their emission can be innovative (e.g., additive manufacturing) [2,3] or more common (e.g., welding, thermal spraying, and thermal engines). Given the very wide diversity of the processes or operations which can lead to the emission of airborne NPs and the broad variety of the industrial sectors concerned, a very large number of workers are potentially exposed to airborne NPs by inhalation [4,5].

Toxicological studies have demonstrated the potential health effects of inhaled NPs [[6], [7], [8], [9], [10]]. From the research conducted over the past 20 years, questions as to the relevance of the “mass and chemical composition” paradigm have emerged. Other determinants that could be considered when analyzing NPs toxicity, including particle size (distribution), shape, and surface characteristics such as surface coatings, have been suggested. However, no consensus has yet been reached in relation to human toxicity assessment [6]. From an epidemiological point of view, the strength of evidence for an impact on health associated with the physicochemical characteristics of NPs remains low [11]. In epidemiological studies, the heterogeneity and level of detail on the data presented can make exposure difficult to assess [4,12]. The lack of a consensus operational exposure assessment strategy that can be implemented across the field in numerous situations and by a large number of occupational hygienists adds to these difficulties.

In this context, multiple strategies to assess occupational exposure to airborne NPs have been developed in various countries. These strategies are proposed by stakeholders and research teams with several specialisms (indoor air quality, atmospheric pollution, occupational hygiene, ergonomics, etc.). As a first step towards a general consensus, three worldwide initiatives to harmonize strategies have been launched [[13], [14], [15]]. According to preventionists in the field, further development will be required to produce clearer and applicable recommendations [16].

In occupational hygiene, an exposure characterization takes into account the factors linked to substances or products, processes, equipment and workplaces, and even to collective or personal protection. As defined by the AIHA, exposure characterization involves gathering information to characterize the workplace, the workforce, and environmental agents. In 2004, the International Programme on Chemical Safety laid the foundation for a common definition of exposure assessment as “*the process of estimating or measuring the magnitude, frequency and duration of exposure to an agent, along with the number and characteristics of the exposed population*”. These additional parameters to the agent to be considered are commonly referred to as “contextual information” [13].

“Work activity” analysis in ergonomics is a complement to the classical industrial hygiene approaches; it serves as a resource to highlight exposure situations. Work activity can be defined in terms of know-how and the resources actually mobilized by workers to achieve the task-objectives set by the company, while also attempting to preserve their health [17]. In the last few years, work activity analysis has significantly contributed to comprehensive risk assessment [[17], [18], [19]]. Building on these studies, measurement and video techniques used to analyze exposure in industrial hygiene [20,21] and work situations in ergonomics [22] should help to increase our knowledge of the determinants of exposure while promoting the development of effective prevention strategies. Some determinants that modulate activities and exposure situations have already been identified by ergonomics approaches. These methods also support calls for a better integration of biological and psychological work activity aspects that may influence exposure and contamination. For these reasons, “work activity” integrates in depth and strategic information for the exposure assessment complementary to the characterization of the workforce.

The objective of this article was to present a critical literature review on the strategies proposed to assess occupational exposure to airborne NPs (ENPs and UFPs). From the information gathered, we formulated recommendations for an operational comprehensive strategy.

## 2 Method

The systematic review was performed on the scientific and grey literature. Documents were selected and analyzed to determine the robustness and practicality of the approaches described; the originality of the approach was also estimated. Each of the steps involved in selection was performed by at least two independent authors, their conclusions were then discussed over the course of several workshops. Given the topic of the review and the expected diversity of the sources of the documents, a simplified approach based on the PRISMA method [23] was adopted.

### 2.1 Sources of information

Scientific literature was sought in the following bibliographic databases: PubMed, Scopus, and ScienceDirect. Articles published from 2000 until May 2021 were retained. To ensure the largest possible initial collection of documents, several keywords were used referring to the two following subjects: *nanoparticles* (including ENPs, NOAA, and UFPs) and *occupational exposure assessment strategy*.

The grey literature was also gathered through websites produced by occupational health research institutes, standardization bodies, or international bodies. These searches were undertaken on the BAuA (German Federal Institute for Occupational Safety and Health), European Agency for Safety and Health at Work (EU OSHA), Health and Safety Laboratory (HSL), Institute for Occupational Safety and Health of the German Social Accident Insurance (IFA), French National Institute for Research and Safey (INRS), Institut de Recherche Robert-Sauvé en Santé et en Sécurité du Travail (IRSST), International Standard Organization (ISO), National Institute for Occupational Safety and Health (NIOSH), Organization for Economic Cooperation and Development (OECD), SafeWork Australia, Netherlands Organization for Applied Scientific Research (TNO), German Chemicals Industry Association (VCI), and World Health Organization websites.

### 2.2 Document selection

Documents were selected based on the following inclusion criteria:

- proposal of a strategy to assess occupational inhalation exposure to NPs based on the knowledge available,
- The strategy must be applicable and intended for application in varied work settings;
- Consideration of metrics and instruments used to characterize exposure;
- Proposal of a strategy relying on previous strategies only if a novel element was presented;
- Documents from the grey or scientific literature resulting from collaboration and based on the scientific literature published in English;
- Full text available online.

The exclusion criteria applied were:

- literature review of strategies presented elsewhere, without novel recommendations;
- Field or laboratory studies aiming exclusively to produce knowledge on exposure or emission;
- Studies describing or relating to control banding approaches;
- Laboratory or field studies investigating the performance of instruments or measurement methods.

### 2.3 Document analysis

The selected documents were analyzed and summarized based on the following descriptors: type of NPs, the objectives of the strategy, and the description of the strategy (presence of a flow diagram or proposal of tiers or a decision-making tool was considered).

Subsequently, the following themes were analyzed more extensively: measurement strategy, how contextual information or determinants related to exposure were described; how working activities were taken into consideration. From the analysis of these themes, a reading grid was filled out for each document selected (Table 1). This reading grid was then used to compare the points of interest and limitations of the various strategies across studies.

**Table 1:** Themes, criteria, and methods used to assess the robustness and operational nature of strategies

Theme	Criterion	Rating		
		0 (None)	1 (Basic)	2 (Advanced)
Measurement strategy	Strength	Not addressed	Personal sampling and/or Electron microscopy and/or Handheld/personal real-time measurement	Personal sampling Electron microscopy Multimetric real-time measurement Aerosol size distribution
	Practicality	No recommendation	Some practical recommendations	Detailed method
Contextual information	Strength	Not addressed or basic information	Detailed information on targets	Exhaustive information
	Practicality	No recommendation	Some recommendations on gathering information	Detailed method
Work activity	Strength	Not addressed	Targeted pattern of work activity	Comprehensive activity analysis
	Practicality	No recommendation	Some advice on methods to describe activity	Detailed method

The “measurement strategy” used to assess exposure in the workplace necessarily involves the selection of the relevant elements to be measured. Several available measurement methods may be used. Integrated sampling (personal or stationary) can be implemented to collect, and subsequently analyze, one (or all) of the reference aerosol fractions in occupational health: inhalable, thoracic, and respirable [24]. Some sampling devices are specifically designed to measure the size distribution of aerosols. In addition, real-time instruments addressing other exposure metrics (number, surface area) or providing information on the exposure profile over time may be included in the measurement strategy. Subsequently, chemical or electron microscopy analysis methods can be applied to the samples collected to determine, e.g., elemental concentrations, particle size, morphology, aggregation or agglomeration state, etc. A method to localize and describe sampling points and the duration of the measurement is generally presented. For the purposes of this article, the information relating to measurement strategies was classified based on three levels: none, basic, and advanced (Table 1). Thus, the description of the strategy may provide no information on measurement techniques; describe a classical basic approach for chemical substances or present an advanced approach, involving multi-metric and real-time measurements, allowing an analysis of the size distribution of aerosols and complementary physicochemical analyses.

“Contextual information” relates to all of the information describing the work environment (including physical, organizational and social aspects), the process (including upstream and downstream steps), and the changes to the work situation which may influence exposure [[25], [26], [27],^13^]. The quality of the exposure assessment and its interpretation depends on the level of detail of this information and how it is exploited [25,28]. The levels defined in Table 1 range from an absence of information (or basic level describing NPs targeted, production sector, etc.), through a description of key information (volume of work area, type of personal protective equipment, work procedure, operation duration and frequency, etc.), up to an almost exhaustive description of the contextual information. In the latter case, the relative information on the work situation must describe all the potential determinants of exposure.

“Work activity” refers to the activity really undertaken by the worker when performing the prescribed task [17,29]. In other terms, the task is defined as an objective to reach, whereas the activity represents the actions and resources (biological, psychological, and collective) mobilized by the worker to complete this task. Thus, considering “work activity” rather than contextual information to be the main factor in occupational exposure constitutes a change of paradigm. The current literature often refers to “worker behavior” [30] as an element of contextual information without considering the actions performed by workers in real work situations or the determinants of those situations [31,32]. For this review, when the physical aspects of work—such as the heart rate or physical effort exerted—were considered when assessing exposure, this information was noted. Thus, the extent to which the work activity was taken into account could range from an absence of consideration, through the consideration of the characteristics of the target activities (such as actions performed by workers, postures, location in the workplace, etc.), up to a detailed comprehensive analysis of the activity (physical strain, operating mode, strategies and actions performed, and workers' risk representations or regulation).

The analysis performed on the three themes mentioned above was used to estimate the strength of the recommendations made in the selected documents, based on how each type of information and the associated details were specified (levels of information).

The practicality of the strategies described was assessed in complement to the strength of the recommendations. Practicality can be considered an operational characteristic. For the three themes, the lowest level of practicality corresponded to a situation where no detail was provided to allow implementation in the field.

For the “measurement strategy” theme, the highest level of practicality corresponded to a presentation of precise information on how instruments were used, on the analytical protocols applied to the samples collected, or on the data analyzed. The ease-of-use of the recommended instruments was also taken into account.

When examining “contextual information”, the highest level of practicality included details on the various steps involved in collecting information, the specific documents required, and the techniques to be used.

Finally, for the “work activity” theme, the highest level of practicality corresponded to a presentation of the steps, observation methods, and activity-related data-processing methods, and of recommendations on how analysis results should be used.

### 2.4 Rating strength and practicality

Each document retained was ranked from the point of view of the strength and practicality of the recommendations made. This ranking was based on the elements listed in Table 1. For each of the three themes, the criteria of *strength* and *practicality* were awarded a score of zero, one, or two. For strength, the ranking was determined as previously proposed [28].

The overall strength and practicality of a strategy were ranked by adding the scores attributed for each of the three themes. Thus, for each document retained, following the analysis of the level of information (strength) and the operational nature (practicality), a rank on a scale of 0 to 6 was attributed.

This ranking was proposed by one author of the article to two other authors from complementary disciplines with a thorough knowledge of the recommendations. The ratings assigned for each theme and criteria to the different recommendations were discussed in line with Table 1. The final ratings obtained were shared to the author panel for verification.

## 3 Results

**Fig. 1:**
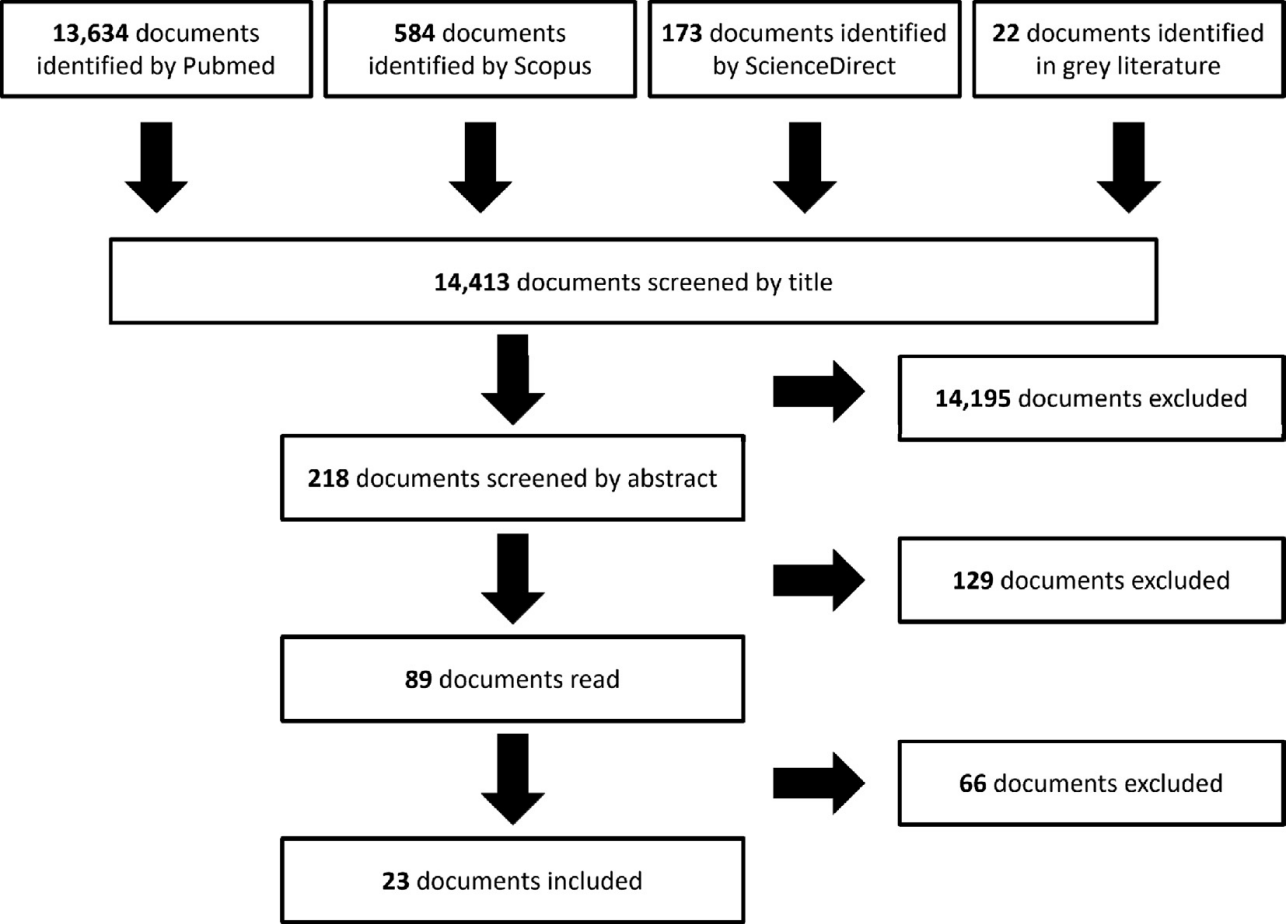
Document selection based on a PRISMA flow diagram.

The initial literature search identified 14,413 documents across all the bibliographic resources consulted. After the selection based on the title, 218 documents were retained. Reading of the abstracts reduced this corpus to 89 potentially relevant documents. Finally, after reading these documents in full, 23 were selected for further examination as they adequately described the strategies. This collection of texts included four standardization documents, seven documents published by occupational health research institutes, nine scientific review articles, one book chapter, and two documents produced by international organizations. Fig. 1 shows how documents were processed and selected for this review.

Table 2 presents the selected documents describing a strategy to assess occupational inhalation exposure to NPs, in terms of the type of NPs targeted and the objectives of the study described. A summary of the various stages involved in the strategy proposed is also presented.

**Table 2:** Documents selected, NPs targeted, objectives, and description of stages

Stakeholders	Reference	Year	ENP or UFP	Document objectives∗	Description of the stages
Standardization bodies	ISO TR 27628 [33]	2007	ENP/UFP	C	n.a.
	ISO TR 12885 [34]	2008	ENP	C, P, GR	n.a.
	BSI PD 6699-3 [35]	2010	ENP	C, P	1: identify the source of emission; 2: basic assessment, measurement at source; 3: detailed assessment; 4: personal sampling
	EN 17058 [15]	2018	ENP	C, P, E	1: initial assessment (products and sources of NPs, existing prevention practices, process/task, and work environment); 2: basic exposure assessment (highlight exposure to NPs); 3: comprehensive exposure assessment (individual and multimetric)
Occupational Health Research Institutes	NIOSH Approaches to safe nano technology [36]	2009	ENP	C, P, GR	1: initial evaluation (identification of emission sources); 2: exposure assessment (multimetric)
	NIOSH (Methner et al) [37]	2010	ENP	C, P	n.a.
	NIOSH Current Intelligence Bulletin 63 [38]	2011	ENP	L	1: collection of the respirable mass fraction of TiO_2_; 2: determination of the sub-100 nm mass fraction of TiO_2_ by ICP and electron microscopy
	BAuA, BG RCI, IFA, IUTA, TUD, VCI [39]	2011	ENP	C, P	1: information collection (estimate the likelihood of the presence of NPs through a risk assessment); 2: basic exposure assessment (detection of NPs during operation); 3: expert exposure assessment (multimetric and multipoint)
	INRS, INERIS, CEA (Witschger et al) ND2355 [40]	2012	ENP	C, P	1: situational study (documentary analysis to determine the presence of nanomaterials); 2: initial assessment (search for potential exposure); 3: *in situ* preparatory visit (approve need to perform a campaign and prepare it), release lab tests; 4: *in situ* measurement campaign (characterize aerosol at source)
	NIOSH Current Intelligence Bulletin 65 [41]	2013	ENP	L	1: identification of tasks potentially leading to exposure; 2: collection of the respirable mass fraction; 3: determination of CNT/carbon fibers by a thermal-optical analysis
	IRSST (Ostiguy et al) R-840 [42]	2014	ENP	C, P, GR	1: preliminary sample (nanomaterial presence, identification of sources and development of the strategy); 2: in-depth investigation (quasi-individual characterization of exposure)
	NIOSH (Eastlake et al) [43]	2016	ENP	C, P	1: collect basic workplace information (workers' practices, tasks and process, information on products and sources of NPs); 2: task-based individual exposure assessment
	IRSST (Debia et al) R-952 [44]	2017	ENP	C	1: preliminary assessment (detection of work tasks generating NPs); 2: in-depth assessment (quantitative and surface exposure assessment)
Laboratories and Research centers	Brouwer et al [45]	2004	UFP	C, P	n.a.
	Woskie et al [27]	2010	ENP	C, E	n.a.
	Lee et al [46]	2011	ENP	C	1: initial assessment (identify emission sources and develop the strategy); 2: main evaluation (qualitative or quantitative assessment of exposure to nanomaterials)
	Ramachandran et al [47]	2011	ENP	C, P	1: basic characterization (describe workers' practices, work environment, and NP sources); 2: exposure assessment (multimetric and multipoint)
	Brouwer et al [13]	2012	ENP	C, P, E	n.a.
	Bekker et al [48]	2015	ENP	C, P, E	n.a.
	Peters et al [49]	2016	ENP	C, P, L	1: basic characterization (collect information on the workforce and workplace: NP sources, tasks, process, etc.); 2: construction of similar exposure group and exposure assessment (concentration mapping, job-task-related measurements, and multipoint and multimetric); 3: interpretation of results (if applicable, compliance with OEL); 4: follow-up and control
	Bressot et al [50]	2018	ENP	C, P	1: collection of basic information about process and material (literature, material and safety data sheet, companies); 2: semi quantitative assessment of exposure (visual observations, inventory of processes and operations, and measurements); 3: exposure assessment for each step in the scenario (multimetric and multipoint)
International bodies	NEDO, RISS, AIST, & TASC [51]	2013	ENP	C, P	n.a.
	OECD No. 55 [14]	2015	ENP/UFP	C, P	1: information collection (products and sources of NPs, preventive measures selected, tasks/processes, and work environment); 2: basic exposure assessment (detecting exposure to NPs and emission sources); 3: expert exposure assessment (individual and comprehensive multimetric in-depth exposure assessment)

∗ **C**: characterization of exposure or emission; **P**: exposure assessment for prevention or verification of control measures implemented; **L**: comparison to exposure limit values; **E**: exposure data for database purposes; **GR**: general recommendations including an exposure assessment strategy, as defined in paragraph 2.3. Document analysis; n.a.: not appropriate.

Table 2 indicates that for more than a decade, numerous stakeholders from institutes specialized in prevention research, from university laboratories and research centers, from standardization, and other international organizations have been proposing recommendations for the occupational monitoring of NPs. However, the types of NPs studied and the objectives were sometimes different, and these considerations had an impact on the strategies proposed. It is interesting to note that a very high proportion (20/23) of the documents reviewed target ENPs, while only one specifically related to UFPs. The authors of the other two articles claim their strategies to be applicable to both UFPs and ENPs.

Two-thirds of the documents (15/23) presented tiered approaches. These articles generally started with a study of the situation (step one often involving simple tools) and then proceeded to an extended field study (involving multiple instruments of varying complexity). The more complex the field study, the more refined the information provided by the exposure results. Tiered approaches are generally represented as a flowchart, which facilitates understanding by the reader, and consequently undoubtedly favor their implementation. In addition, some steps indicated in these flowcharts are directly conditioned by the results of the previous steps (measurements, observations, scientific, and technical information). Several criteria can be used to draw conclusions on the acceptability of the exposure measurement depending on the measurement results. Some of these criteria involve comparing results based on background measurements [52,15], whereas others favor comparisons with nonregulatory limit values [49].

**Fig. 2:**
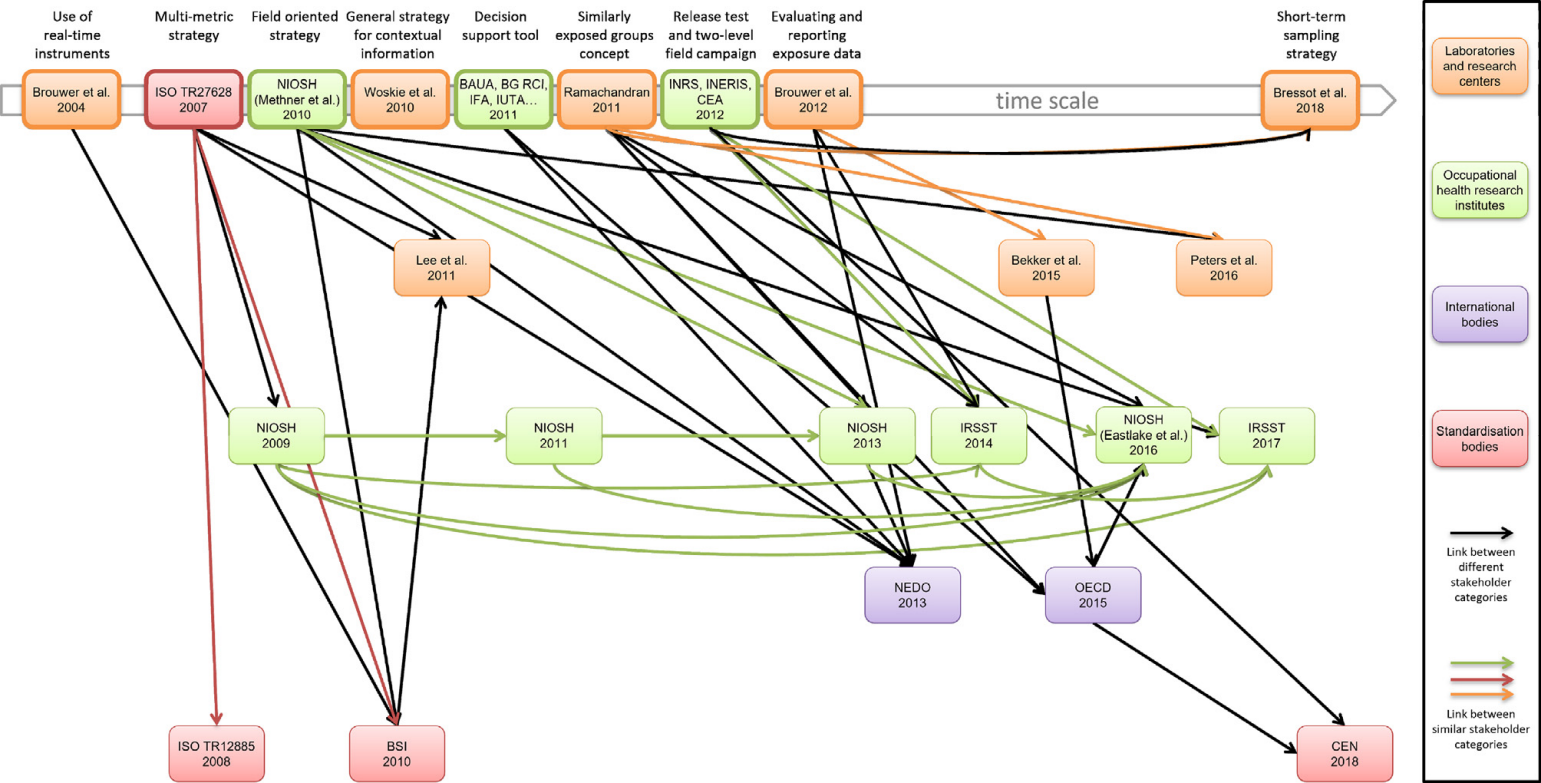
Mapping of selected strategies: publication date, key elements, and links identified.

The strategies included in the literature review listed in Table 2 included one or more steps: one (eight documents), two (seven documents), three (five documents), or four (three documents). The fourth step was often a complementary analysis level. By formalizing the links between the different strategies described, the various strategies were shown not to be completely independent. Fig. 2 presents the links identified between the 23 documents included in the literature review. The principal axis shows documents describing strategies that were qualified as “novel”. Above each strategy, the reasons for this qualification are summarized in a few words.

The earliest document selected for this review was published in 2004 [45]; it relates the first exploration of a strategy to characterize exposure based on a multimetric approach. In a situation where workers were exposed to UFPs (welding), this strategy revealed the need to integrate both real-time measurements and sampling for the subsequent analysis. A short time later, an international standardization document focusing on air quality in the workplace (ISO/TC146/SC2) was developed by a technical committee. This document included guidelines for the characterization of occupational exposure, discussed the various metrics that could be considered, and described the appropriate measurement instruments along with their advantages and disadvantages [33]. This second founding document is devoted to assessing exposure and to the need to combine a classical exposure assessment approach with a more innovative approach relying on the use of various instruments, such as direct-reading devices, which were still little used at the time of publication. In addition, the authors formulated general recommendations on how the background aerosol should be taken into account (deferred or parallel measurement, electron microscopy observation). The first ISO technical report (2007) [33] was a significant source for the BSI English-language version of the standardization document in 2010 [35]. After 2010, several articles were published by authors based in the Northern Hemisphere [13,46,37,47]. The first steps towards the European harmonization were taken in Germany by a working group including several occupational health-focused institutes or reference organizations: BAuA, BG RCI, IFA, IUTA, TUD, and VCI. This working group developed a three-tier approach [39] which can be extensively used by companies. In France, a similar working group was set up in 2012 with representatives of INRS, INERIS, and CEA [40]. It was recommended to integrate laboratory tests as part of a preliminary control banding type assessment (corresponding to dustiness tests). Another novelty of this strategy was that it was developed for two categories of users: experts in exposure and occupational hygienists. It should be noted that only one strategy by Ramachandran et al [47] integrates the notion of similar exposure groups (SEGs). Other authors underlined the need to integrate contextual information which may influence exposure [27,13]. A more precise description of the contextual information and work activities that are most relevant to NPs (description of operations, characteristics of the work environment, tasks, etc.) only emerged more recently [48,15,43,14].

The most recent documents (EN 17058 [15], NIOSH [43], and OECD [14]) also emphasize the operational nature of strategies. The proposals emerging from Northern America (NIOSH, IRSST) have pronounced specificities, in particular with regard to the number of steps—the strategies are generally shorter—and the number of field measurement techniques deployed. These proposals tend to be more directly operational than the European proposals (BAuA, BG RCI, IFA, IUTA, TUD, VCI [39] or Brouwer et al [13], Bekker et al [48], and Bressot et al [50]), which focus heavily on analysis techniques and instrumentation.

Finally, the concept of “short-term sampling” defined by Bressot et al [50] emerged very recently. This qualitative approach aims to characterize emissions and highlights the advantage of performing short-duration sampling (from a few seconds to a few minutes) for observational purposes and can help identify exposure situations resulting from short-lived events.

**Fig. 3:**
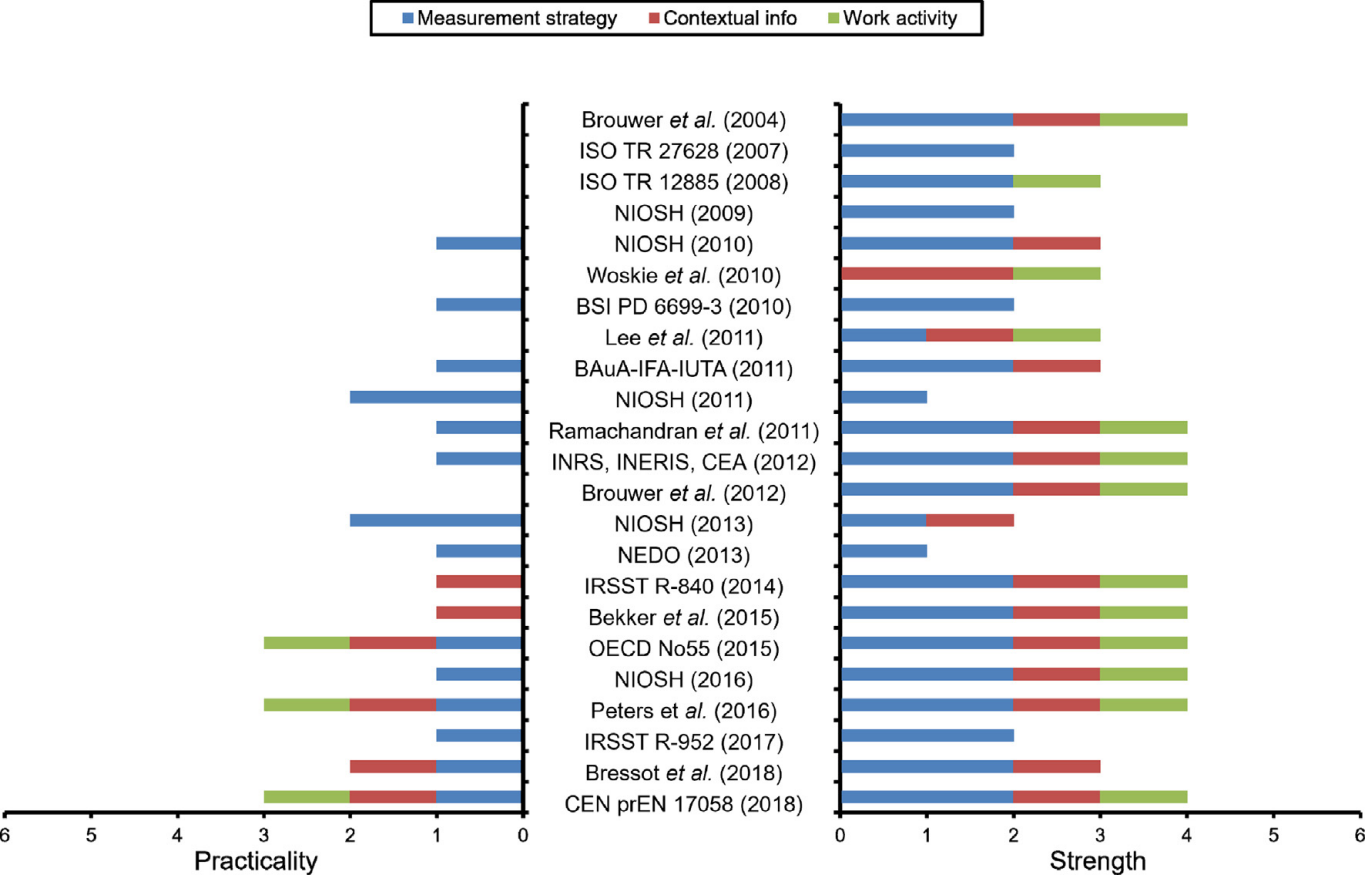
Practicality and strength of the selected strategies based on measurement strategy, contextual information, and work activity.

For each document, the robustness and practicality, whether based on “measurement strategy”, “contextual information”, or “work activity” were determined (Fig. 3). In terms of the strength of the different strategies, it can be observed that most strategies were awarded the top score of 2 from the point of view of “measurement strategy”. In contrast, they were not so well ranked in terms of “contextual information” or “work activity”. Thus, only Woskie et al [27] was ranked 2 for “contextual information”. The most striking result that emerged from this, Fig. 3, is the very low degree of practicality of most strategies, whatever the theme examined, except for a few recent documents such as EN 17058 [15] or OECD [14]. In complement, strategies including in depth measurement approaches were only presented from 2004, and the significance of “contextual information” has only been taken into consideration since 2010.

Almost all the documents selected neglected to consider the “work activity”. In the best case, some documents mentioned the importance of integrating the “work activity” using various terms: activity description [48], record of workplace activities [14], document workplace activities [43], or description of actions performed by workers [15]. Only a single document indicated that postures and positions adopted by the workers in their working environments should be reported [27]. Ostiguy et al [42] refer to risk perception, movements, and the working methods used by workers without providing further detail. Approaches such as those described in EN 17058 [15], NIOSH [43], Peters et al [49], and Ramachandran et al [47], all of which are written from the point of view of prevention, place greater emphasis on the work performed.

## 4 Discussion

The results indicate that a wide variety of strategies are currently used to assess occupational exposure to airborne NPs. This diversity can be explained by the following differences.

- The objectives for which the strategies were developed;
- The stakeholders developing the strategies and their respective fields of expertise;
- The target NPs (ENPs or UFPs);
- The measurement instruments and analysis protocols available;
- The aerosol fractions taken as reference, as well as the target metrics (number, surface area, or mass).

Documents produced by standardization or international organizations or resulting from country-specific harmonization initiatives— such as the proposals developed in Germany and France—generally attempted to present a cross-disciplinary consensus. The OECD and the CEN have also significantly contributed to developing harmonized strategies.

In addition, the documents studied systematically indicated that a high level of expertise is necessary to achieve a comprehensive exposure assessment. This expertise is mainly linked to the implementation of the “measurement strategy”.

### 4.1 Measurement strategy

The “measurement strategies” described often require the implementation of complex measurement instruments, which in some cases are not well adapted to the constraints of the fields observed [13,14]. In addition, physicochemical analyses may be required, involving protocols that are not always available or validated. This state of affairs hinders the accrual of information [53], and, consequently, hygienists often lack the data required to become fully conversant with the risks linked to NPs.

In our analysis, we noted significant differences in terms of instrumentation between the earliest strategies described [45,33,54] and more recent ones [15,43]. Indeed, although the metrics were often similar, some instruments have evolved and are now more appropriate for use in the field (extended battery life, smaller size, portable by workers) [55].

### 4.2 Contextual information

The importance of integrating this type of information in parallel to the measurement strategy was already highlighted in the earliest published document [45]. This contextual information was recognized during an international workshop devoted to the harmonization of the strategies to measure exposure to ENPs [13]. A call for contextual information was also included in a document published by the OECD [14] and in the European Standard 17058 [15].

This information could be complemented by other factors potentially influencing exposure, present at the level of the workstation or as the result of organizational, design, or company-operating decisions [56]. Contextual information relating to the production sector, the geographical area where the company is located (neighboring environment, sociocultural patterns, etc.), or preventive policies [27].

In an overall risk-analysis approach, the work performed must also be considered through the implementation of work analysis techniques developed in the field of ergonomics [17].

### 4.3 Work activities

Both the methods used to analyze activity and the level of detail of the descriptions of the activity to be observed require further development for situations leading to potential exposure to NPs. This development is necessarily specific to each work situation and is distinct from the contextual information. Exposure is commonly defined by the contact between a worker and a pollutant [57,58]. In this sense, the factors governing the activity and the workplace relative factors should be described by the contextual information, whereas the activity itself constitutes the worker activity. Work activity stands for the real work undertaken, leading to real exposure of the worker in a specific situation.

An example can be given to clarify the distinction between “contextual information” and “work activity” from a recent work in a rubber industry [59]. The “contextual information” would be relative to the following information. A 25 year old worker (1.60 m and 55 kg) is requested to weigh 30 times 10 kg of powders from 25 kg bags. These bags contain zinc borate and carbon black. This work takes place in standard industrial premises with open metallic curtains, with neither general ventilation nor local exhaust. The worker was wearing short sleeves and nonprotective cotton work clothes and no respiratory protection. There are no rules which require the wearing of personal protective equipment. No co-activity was observed apart from the presence of a mixer and a big bag. The worker indicates a fear of becoming ill. The “work activity” would be summarized as follow. The worker changes the empty bag, brings a new bag, opens the bag at the top, handles powders from a scoop or a 25 kg bag, closes the weighed bag and transfers it into a tray, and finally cleans the workstation with a broom. The worker sometimes holds his breath and tries to deposit the powders gently to avoid resuspension. The worker's physical strain can be described as quite heavy to very heavy (which can increase the exposure level).

Work activity analysis helps identify the exposure determinants and how work activity influences exposure situations. Based on the information it provides, transformations of exposing work activity determinants can be proposed as part of attempts to eliminate, or at least reduce, the hazardous situations. These holistic methods also take into account the physical intensity of the work.

Approaches that involve various stakeholders across the company (workers, managers, and preventionists) help make all these categories of workers aware of exposure and promote sharing of know-how and knowledge [20]. The stakeholders themselves are encouraged to formulate hypotheses on the sources of exposure. For several years already, industrial hygiene has promoted the use of video recordings synchronized with real-time measurements when aiming to assess exposure to chemicals or physical hazards [20]. In their analysis, Beurskens-Comuth et al [21] clearly showed that synchronizing several measurement signals with the video recording could be useful to simultaneously observe various points, e.g., at the level of the worker's personal breathing zone and in terms of the potential sources in the environment near to or at a distance from the worker. Thus, video recordings of work activity—by making it easier to gather contextual information about work situations—can help identify events leading to variations in exposure levels, as well as variations in determinants. In these conditions, the work activity itself becomes part of the exposure assessment strategy.

## 5 Recommendations for assessment of exposure to airborne NPs

The strategy proposed is not restricted to an expert approach and centers around two operational assessment stages: preliminary and in-depth. A preliminary assessment can be made to refine the hypotheses on the situations leading to exposure that should be the focus of investigation during the in-depth assessment stage. The in-depth assessment can then be organized around the assembled information, integrating a first level of contextual information and activity analysis.

These recommendations are formulated as part of a cross-disciplinary approach [16] implemented in France involving a group of authors from different fields: aerosol metrologists, ergonomists, epidemiologists, and occupational hygienists.

### 5.1 Preliminary assessment

At the preliminary stage, the following information should be collected: potential exposure, type of activities, materials and processes implemented, risk assessment and work procedures, how the company functions, work environment, work organization, ventilation, and work-specific and preventive equipment. In parallel, directed interviews relating to the exposure hypotheses should be conducted with the stakeholders in the company, in particular with the representatives from management, occupational health professionals, and workers. The information gathered will help to identify potential exposure situations and representations. Finally, a simple observation of working situations potentially leading to exposure should be associated with real-time measurements. Some stationary measurements of the number and mass concentrations at both the nanoscale (or submicronscale) and the microscale could be carried out in the workplace and outdoors to map the sources of particles. These results will provide initial information on exposure, which can subsequently be used to refine an appropriate in-depth assessment.

### 5.2 In-depth assessment

The in-depth assessment should focus on personal exposure, based on a two-level strategy involving classical and advanced methods (Table 4). The analysis will require a strategy including integrated sampling and real-time measurement techniques. Through this approach, airborne particles with sizes ranging from a few nm up to 10 μm can be targeted to cover the wide range of possible sizes of NOAA. In parallel, care should be taken to gather macroscopic contextual information and contextual information on the work situation (organizational factors related to the work, the management method, collaborative work, task distribution, safety rules, etc., as well as technical aspects such as processes and materials, collective and personal protection, etc.) and information on the work activity (using video recordings and physiological measurements). Table 3 illustrates contextual and work activity-related information to be considered from the seminal works [[25], [26], [27],^13^]. The work situations to be targeted for the in-depth assessment can be based on typical or potential exposure situations to constitute SEGs. SEGs should be developed from the information gathered during the preliminary analysis and implemented during the in-depth analysis.

**Table 3:** Contextual information and work activity patterns to take into account for the in-depth assessment

Work activity	Activity stages (successive actions), operating mode, postures and positions, logic of action, physical strain, psychological strain, temporal continuity and occurrence, events and variability, contact and contamination, know-how, and risk-taking		
Contextual information	Work situation level	Workers	Age, height, weight, training, career paths, experience, values and representation, and inter and intra individual variability
		Agent	Categories and sources of NPs
		Technical system	Work environment, process, tools, engineering control, protective equipment, and atmospheric conditions (air change rate, room volume, air flow pattern, and atmospheric pressure)
		Work organization	Task-objective, procedure, instruction, function, production operation, work team, working rates and hours, co activity, maintenance, and cleaning
	Company level	Culture, policies and health and safety system, risk management, and assessment document	
		Production devices, design of production devices, technological process, ongoing projects, and R&D	
		Geographical location, production sector, production, and characteristic (size, operation, history …)	
		Regulation, agreement, and standard	

As a first step, particle concentrations based on both number and mass concentrations should be monitored in real-time. The mass concentration must target the conventional respirable fractions to ensure that results are comparable to classical integrated sampling results. In parallel, the number concentration must cover the nanometric scale (<1 μm). For classical approaches, different size classes, including nanometric scale, should be sampled by integrated sampling methods. Advanced approaches allow the implementation of video observation of the work activity alongside real-time measurement and integrated sampling techniques to refine the characterization of the nanoscale particles, in terms of nature, physiological intensity, and an estimated deposited dose.

Unlike physicochemical analyses, the aerosol measurement techniques described in Table 4 are not specific to a single particle type. Therefore, to provide elements relating to how worker's activities influence exposure, the background aerosol must be characterized [60]. Real-time and integrated sampling techniques, using the same metrics, must be simultaneously applied in the workers' personal breathing zone and for background monitoring.

**Table 4:** Indicators for the in-depth assessment of exposure to nanoparticles by inhalation, integrating aspects related to work activity

Integrated sampling		Real-time measurement
Inhalable and respirable fractions. Subsequent analyses: Gravimetric analysis, Chemical analysis specific to the pollutant (ICPMS, carbon, XRD, etc.)	**Classical**	Temporal profiles of number and mass concentrations
Sampling of the different size classes. Subsequent analyses: Gravimetric analysis (mass distribution of particles), chemical analysis (elementary distribution)	**Advanced**	Temporal number and mass concentration. Size distribution profiles
Electron microscopy. Subsequent analyses: morphological observations, size (or size distribution) of particles, indicative composition (EDX)		Video observation of the work activity
Estimation of the lung-deposited dose		Physiological measurements (e.g., heart rate and respiratory rate): estimation of the physical intensity
Measuring background aerosol (spatial, temporal, and physicochemical analysis)		

Concurrently, it is recommended to use video recording—after obtaining the worker's consent—to create a visual link with real-time measurements when attempting to identify particular situations or work activities leading to exposure. To better integrate the physical intensity of work activities and study how it influences exposure, the heart and respiratory rate should also be recorded in real-time.

In the second step, cleaned and formatted measurement data must be processed [59]. Various results can be extracted from real-time data: from time series to more integrative representations (e.g., boxplot). By integrating activity analysis (e.g., using video-exposure monitoring) and background levels, variations in exposure can be documented, thus producing a more refined exposure assessment. A temporal scale of interest for the exposure analysis should be chosen to make variations visible with their determinants. By associating the physical intensity of the work performed (e.g., through real-time measurement of the heart and respiratory rate) with airborne NP concentrations, potential deposits in the airways can be estimated using deposition models [61,62]. A complete representation of exposure can be obtained by adding physicochemical characterization of the NPs, for which several classical protocols are available (e.g., in the MDHS (HSE), MétroPol (INRS), NMAM (NIOSH), and IRSST factsheets). The advanced integrated sampling methods listed in Table 4, meanwhile, require further development.

In complement, it is strongly recommended that the results be presented to a range of stakeholders in the company, to encourage discussion of the work situations leading to exposure, and to develop preventive actions as a group. The real-time measurement data and video recordings of work situations can be used as supports for these discussions [22]. In this context, an across-the-board understanding of the situations leading to exposure to NPs, and the means to control them, becomes possible.

## 6 Conclusion

This article presents a critical review of the scientific and grey literature on the strategies proposed to assess occupational exposure to airborne NPs. Twenty-three documents published between 2004 and 2021 were analyzed to determine the relevance and practicality of the recommendations described. These considerations are presented here according to three criteria: “measurement strategy”, “contextual information”, and “work activity”. The 23 documents analyzed, produced by various stakeholders, revealed heterogeneous levels of relevance for the three criteria assessed; and a large majority of the strategies reviewed scored very low for practicality, whatever the criterion examined. Based on this analysis, it emerges that strategies are gradually evolving to integrate the analysis of worker activity with exposure measurements in the context of exposure to NPs.

In the final part of this paper, several recommendations are made for an integrated strategy to assess exposure to airborne NPs. The suggested strategy relies on classical measurement methods, associated with contextual information and workers activity observations. It can be applied to all types of situations, processes, and particles, whether manufactured or unintentional, and is independent of the particles' chemical composition, morphology, and size. The strategy proposed is intended for use by the various stakeholders involved in prevention in the field and should be compatible with the integration as part of typical occupational health interventions.

## Conflict of interest

The authors declare no conflict of interest.
